# The necroptosis cell death pathway drives neurodegeneration in Alzheimer’s disease

**DOI:** 10.1007/s00401-024-02747-5

**Published:** 2024-06-09

**Authors:** Sriram Balusu, Bart De Strooper

**Affiliations:** 1 Laboratory for the Research of Neurodegenerative Diseases, VIB Center for Brain and Disease Research, 3000 Leuven, Belgium; 2 Leuven Brain Institute, KU Leuven, 3000 Leuven, Belgium; 3 UK Dementia Research Institute at UCL, London, WC1E 6BT UK

## Abstract

Although apoptosis, pyroptosis, and ferroptosis have been implicated in AD, none fully explains the extensive neuronal loss observed in AD brains. Recent evidence shows that necroptosis is abundant in AD, that necroptosis is closely linked to the appearance of Tau pathology, and that necroptosis markers accumulate in granulovacuolar neurodegeneration vesicles (GVD). We review here the neuron-specific activation of the granulovacuolar mediated neuronal-necroptosis pathway, the potential AD-relevant triggers upstream of this pathway, and the interaction of the necrosome with the endo-lysosomal pathway, possibly providing links to Tau pathology. In addition, we underscore the therapeutic potential of inhibiting necroptosis in neurodegenerative diseases such as AD, as this presents a novel avenue for drug development targeting neuronal loss to preserve cognitive abilities. Such an approach seems particularly relevant when combined with amyloid-lowering drugs.

## Introduction

Alzheimer’s disease (AD) may have multiple primary etiologies (genetic, sporadic, environmental, and infection) [15, 68, 90] but is defined by a common characteristic neuropathology and symptomatology, including extracellular deposition of amyloid plaques, intracellular deposition of hyperphosphorylated Tau tangles, neurovascular changes, reactive gliosis, cognitive impairment, granulovacuolar degeneration (GVDs), and neurodegeneration [6, 86, 153]. AD is estimated to affect around 6.9 million people in America alone and is expected to triple by 2050. The total lifetime cost of care for someone with dementia was around $400,000 in 2023 (Alzheimer’s facts and figures 2024).

Loss of functional synapses and neurons is a defining feature of neurodegenerative diseases (NDDs). The correlation between cognitive decline, neuronal cell death, and neuropathological alterations, especially Tau pathology, is relatively well established in AD [62]. However, it remains debated whether neurons die and degenerate by a programmed cell death mechanism or become atrophic and cleared by phagocytic microglia, as some speculate [156]. The question also remains whether neuronal cell death spreads over the brain in a predictable pattern similar to amyloid deposition [162], Tau pathology [22], or granulovacuolar degeneration (GVD) [163] or whether it occurs randomly. How do neurons manage to survive during the lengthy prodromal stage of the disease while all pathology is accumulating—but no cognitive decline is yet observed. Is there a tipping point when neurons begin to degenerate?[150]. It also remains unclear how much neuronal pathology has to accumulate before patients start to show clinical symptoms.

Neurons are regarded as long-lived cell types [104]. The literature suggests that neurodegeneration is often associated with the accumulation of toxic aggregates of hyperphosphorylated Tau (pTau) in the excitatory neurons [44, 46, 48], and that accumulation of pathological Tau is closely correlated with synaptic dysfunction and dementia [47, 112, 126, 140]. Programmed cell death (PCD) is an intricately regulated process involved in tissue homeostasis maintenance of multicellular organisms [47]. Several pathways are at play, such as apoptosis, pyroptosis, ferroptosis, and necroptosis [183], and crosstalk between these pathways is referred to as PANoptosis, characterized by the presence of the PANoptosome [128].

The clinical manifestation of AD correlates well with the degree of synaptic and neuronal loss in the hippocampus and cerebral neocortex. Selective loss of pyramidal excitatory cell populations in the hippocampus and cortex, coupled with deficits in neuronal circuits, is thought to lead to cognitive impairment characteristic of AD. However, cognitive impairment is a relatively late symptom of the disease, suggesting that extensive damage occurs already preclinically. Examination of postmortem AD brains indicates indeed significant reductions in brain volume and the number of neurons [5, 6, 127, 145, 175] as supported by stereological and Isotropic fractionator techniques that allow accurate estimates of the absolute number of neurons in asymptomatic-AD and severely demented AD cohorts. Neuronal density quantification using these unbiased stereological methods revealed a > 50% loss of neurons in the hippocampus and cerebral cortex of demented patients with AD but not in asymptomatic-AD brains [5, 64, 149]. Post-mortem human tissue offers valuable insights into pathological processes; however, these tissue samples represent only end stages within an ongoing disease continuum. The fundamental limitation of endpoint neuropathological analysis is that it solely captures the snapshot of neuronal loss, without elucidating the transient alterations and ephemeral neuronal loss, including the active clearance of dead neurons by resident immune cells.

The Tau pathology precedes neuronal cell death and shows a rather robust correlation with neurodegeneration and cognitive decline in AD. This contrasts with the fact that Aβ pathology does not correlate well with cognition [62]. Pathological buildup of Tau protein is observed in excitatory neurons in specific brain regions, including the locus coeruleus [60], cholinergic basal forebrain [22, 165], the entorhinal cortex [17, 22], the subiculum [22], the hippocampus [21, 22], and the cortical areas. Frequently, the cells harboring pathological Tau also exhibit deficits in autophagic and endo-lysosomal systems [134]. When neurons undergo cell death, intracellular neuronal tangles transition into extracellular ghost tangles characterized by stability, protease resistance, and non-immunogenicity. These attributes enable the longitudinal tracking of deceased neurons through the persistence of these tangles over time. Assuming a one-to-one correspondence between ghost tangles and deceased neurons, if tau-induced neuronal demise primarily occurs via ghost tangle formation, it is reasonable to infer that the abundance of ghost tangles mirrors the extent of neuronal loss. Consequently, quantifying neuronal density in AD patients should theoretically account for both ghost tangles and healthy neurons, aligning with the total neuronal count observed in healthy controls. However, such an assumption leads to a significant underestimation of neuronal depletion in AD, as evidenced by a notable incongruity in neuronal density quantification. Specifically, the loss of neurons in the hippocampus, estimated to exceed 50%, exceeds the prevalence of extracellular neurofibrillary tangles, estimated at approximately 8% [88]. This incongruence suggests that tangle formation alone may not be the sole driver of neuronal cell demise.

This review summarizes the recent evidence that necroptosis, one of the major neuronal cell death pathways in AD, is linked to Tauopathy. We will speculate on which factors might induce neuron-specific activation of the necroptosis pathway and discuss the possibility of targeting this pathway to impede neuronal loss and to counteract neurodegeneration in AD.

## Apoptosis in Alzheimer’s disease

Apoptosis and necroptosis are two distinct modalities of regulated cell death. Developing neurons readily engage the apoptosis cell death pathway but mature, post-mitotic neurons employ various, redundant, strategies to prevent apoptosis (reviewed in [85, 137]). They are, for instance, typically resilient to intrinsic apoptosis triggered by mitochondrial damage [85], which may ensure their long-term survival. Despite this, previous studies have argued that apoptosis is activated in the human AD brain. These claims are primarily based on studies showing caspase-3, -6, and -8 upregulation in AD brain using gene expression studies or immunohistochemistry. However, the characteristic typical morphology of apoptotic bodies is rarely observed (1–3 neurons per million) in AD brains [76]. Caspase-3 is a key executionary protease in the apoptosis pathway, and once activated, it destroys many structural and regulatory proteins in the cell, leading to cellular demise. However, caspase-3 immunoreactivity in AD was detected mainly in GVD bodies and in Tau tangles, and Caspase-3 has been linked to the cleavage of a truncated form of Tau at position 421 in the C-terminus [29, 76, 94, 103, 114, 154]. Although Caspase-3 is activated in AD, the reason why it seems to cleave only a select number of substrates (e.g., Tau and APP) remains unclear [54, 139]. In parallel with findings from human studies, investigations conducted on an experimental animal model (rTg4510 mice) using 2-photon imaging demonstrated that only a small subset of neurons exhibited caspase positivity [152]. Interestingly, despite the presence of activated caspases, the caspase-positive cells did not undergo apoptosis over time. In addition, histochemical assessments unveiled evidence of caspase-cleaved Tau, yet no TUNEL-positive cells or apoptotic morphologies [152]. Hence, the lack of typical apoptotic blebbing morphology in degenerating neurons, the exceedingly low frequency of caspase-3 positive neurons (which does not explain the substantial neuronal loss) [10], and the presence of caspases within Tau tangles [8, 50, 139] all imply alternative functions of caspases in AD, rather than their involvement solely in apoptotic cell death [26, 29, 32, 52].

## Necroptosis in Alzheimer’s disease

**Fig. 1:**
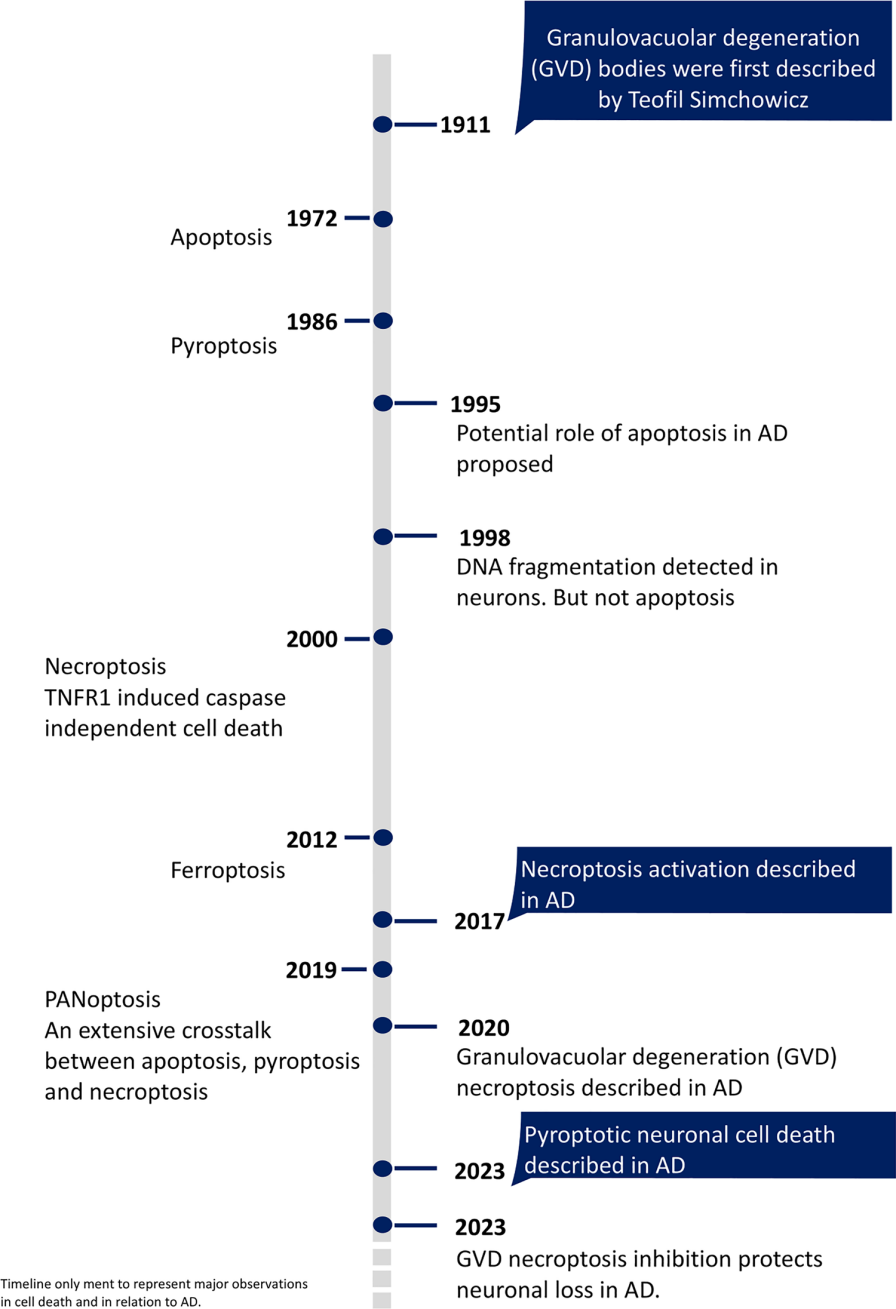
Discovery of cell death pathways in relation to observations in Alzheimer’s disease. The discovery of the different cell death mechanisms is indicated on the left side of the time bar. On the right side, significant observations in Alzheimer’s disease are indicated

**Fig. 2:**
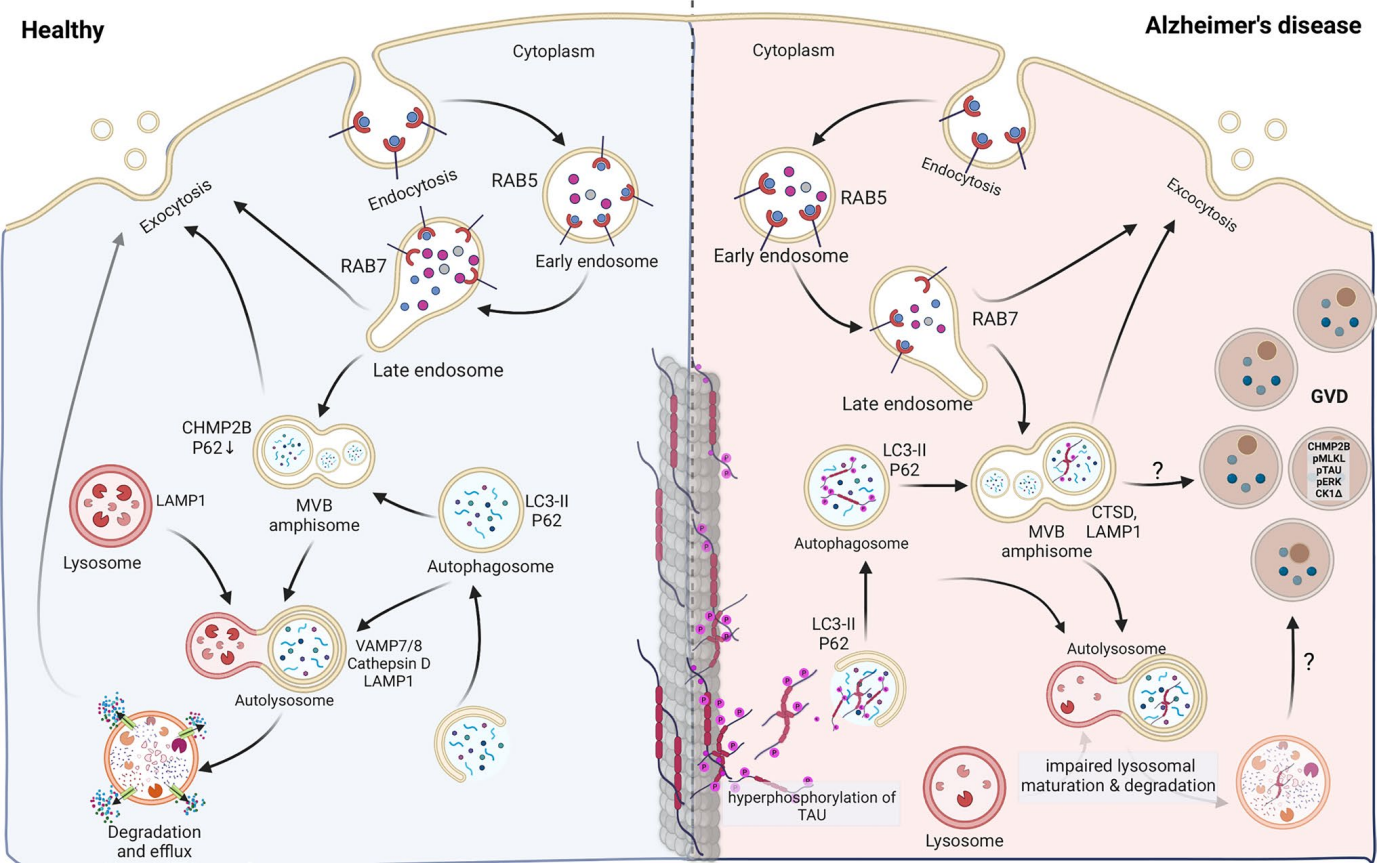
Link between endocytosis, autophagy, GVD, and necroptosis in Alzheimer’s disease. A schematic depiction of the interplay between the autophagic and endocytic pathways in healthy conditions (left side) and in Alzheimer’s disease (right side) in relation to granulovacuolar degeneration bodies (GVDs) pathology. In Alzheimer’s disease, intracellular pathological aggregates such as Tau enter the autophagy and endo-lysosomal system for degradation. Tau aggregation can disrupt proteostasis, intracellular trafficking, and function of this system. The proteolytic capacity may also be inadequate to efficiently degrade the toxic aggregates, increasing demand on the lysosomal system to break down pathological Tau. Impaired endo-lysosomal function and overloading of the lysosomal system can result in the accumulation of undigested cytosolic cargo in GVD. Representative protein markers from every organelle are indicated

**Fig. 3:**
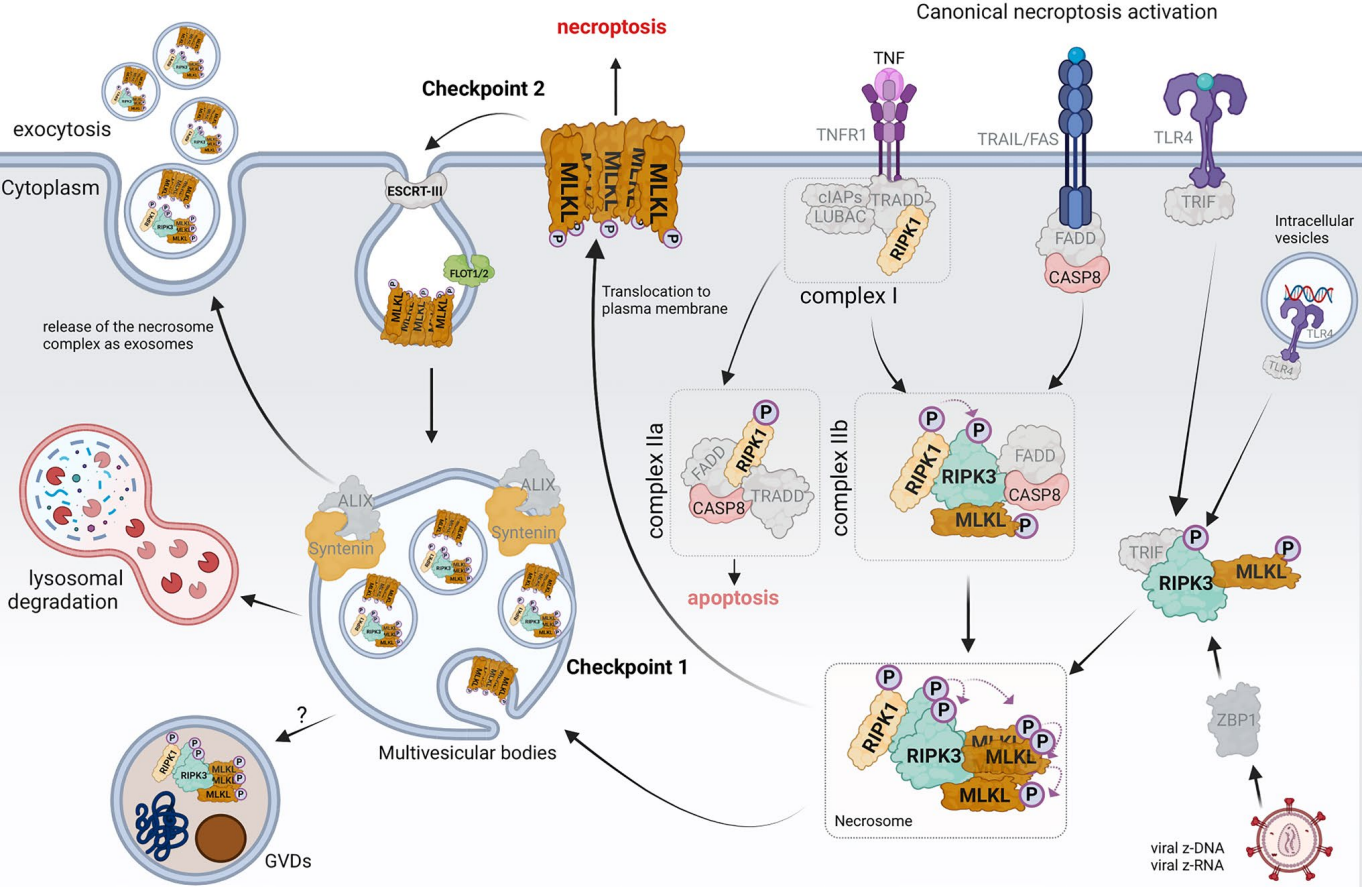
Necroptosis checkpoint mechanism in neurons in Alzheimer’s disease. The canonical necroptosis pathway is initiated by the binding of ligands to cell surface death receptors such as TNFR1. Upon TNF binding to TNFR1, a transient intracellular complex (referred to as complex I) forms, recruiting adaptor proteins like TRADD, RIPK1, and other key signaling molecules involved in TNFR1-mediated NF-kB signaling (not shown in the image). However, dysregulation of complex I leads to RIPK1 activation through phosphorylation at S166. This results in the formation of two distinct cytosolic complexes, complex IIa, and complex IIb, which facilitate RIPK1-dependent apoptosis or necroptosis, respectively. Complex IIa comprises FADD, CASP8, and RIPK1, promoting CASP8 activation, which cleaves pro-caspase-3 into active, cleaved caspase-3, initiating the apoptotic cell death pathway. When apoptosis is impaired or inhibited, activated RIPK1 binds to RIPK3 to form complex IIb. Phosphorylation of RIPK3, either by RIPK1 or through autophosphorylation, leads to MLKL phosphorylation, causing its translocation to the plasma membrane and induction of necroptosis by disrupting plasma membrane integrity. Moreover, membrane-bound TLR4 can trigger necroptosis either upon LPS binding or by interacting with cytosolic viral nucleic acids via TRIF and can directly bind to RIPK3, bypassing RIPK1 to induce necroptosis. Cytosolic viral Z-DNA or Z-RNA can also prompt necroptosis by binding to Z-DNA-binding protein 1 (ZBP1), mediating RIPK3-dependent necroptosis. The phosphorylated MLKL oligomerizes to execute cell death. In Alzheimer’s disease, internalized necrosome complexes can accumulate inside neuronal somas as GVDs due to impaired endo-lysosomal systems (checkpoint 1). Increased levels of oligomerized pMLKL in the cytosol lead to its translocation to the plasma membrane. The oligomerized pMLKL on the damaged plasma membrane can be removed through endocytosis mediated by flotillin and ESCRT-III, followed by degradation in lysosomes, or by exocytosis mediated by Alix and syntenin in the form of exosomes (checkpoint 2)

Necroptosis represents a programmed type of necrotic cell demise. It results in the lysis of the cell and, in contrast to apoptosis, activates inflammation. Necroptosis is induced by the activated necrosome complex comprising phosphorylated forms of receptor-interacting serine/threonine-protein kinases (RIPK1, RIPK3) and phosphorylated mixed lineage kinase (MLKL). Activation of death receptors such as TNFR1, FAS, and TLR4 by their cognate ligands facilitates necroptosis activation. Inflammatory signals primarily induce the canonical necroptosis pathway, typically under conditions where apoptosis is impaired or inhibited (Fig. 3) [86, 129]. Interest in the role of necroptosis in neuroinflammatory and neurodegenerative conditions got traction in the last decade. Necroptosis occurs in AD (Fig. 1) [11, 28, 86], Parkinson’s disease (PD) [38, 100, 125], multiple sclerosis (MS) [133] and amyotrophic lateral sclerosis (ALS) [138]. However, the extracellular and intracellular factors that trigger necroptosis in neurons in NDDs remain elusive, which probably reflects the uncertainties with regard to the upstream or downstream role of inflammation in those different disorders (Fig. 2). A strong caveat, as with the apoptosis studies, is that the mere presence of biochemical markers of necroptosis in tissues does not necessarily demonstrate that necroptosis is effectively occurring, as key mediators of necroptosis may have multiple functions and be involved in extensive crosstalk with other cell death pathways [34, 35, 102, 123]. Moreover, as we will discuss, several late checkpoints in the necroptosis pathways might delay or stop entirely the perforation of the cell membrane and dismissal of neurons [41].

In AD, necroptosis is now unequivocally demonstrated to be at least partially responsible for the dismissal of neurons, and this claim is based on experiments in patient brain samples, xenografted human neurons, and transgenic mouse models of AD [11, 28, 86, 87]. Earlier findings suggested the activation of necroptosis in neurons and, to some degree, in microglia using postmortem AD brains [28]. However, this study primarily relied on the expression of total MLKL rather than the activated form of MLKL (pMLKL). This is insufficient as induction of necroptosis requires the formation of complex IIb (Fig. 3), and this is characterized by the phosphorylation of RIPK1, RIPK3, and MLKL [28]. Using well-established control, AD and pre-AD (pre-symptomatic) brain samples, it was later demonstrated that the activated necrosome complex is exclusively expressed in neurons in brain regions known to be susceptible to neurodegeneration (hippocampal subfields, subiculum, entorhinal cortex, temporal cortex, hypothalamus, amygdala, and frontal cortex) [86].

Furthermore, the necrosome complex was localized in specialized cytoplasmic compartments in the neurons known as GVD bodies [86], which seem to sequester the necrosome complex to avoid immediate cell death execution, which would explain the abundant presence of necroptosis markers in these GVD in AD brains [11, 86] (Fig. 3). The GVD-necrosome complexes are correlated positively with TAU pathology (Braak staging) and are correlated inversely with neuronal cell density in the hippocampus region and the late affected frontal cortex layers [86]. Neurons with GVD and positive for necroptosis markers are notably abundant in the brain, with over 50% of the pyramidal neurons exhibiting activation of necroptosis markers in the hippocampus of AD brains. While these studies in the human brain underscored the importance of necroptosis in AD, they do not allow us to conclude that the lost neurons were dismissed by necroptosis or another cell death pathway. We, therefore, used a xenograft model of AD [11], exposing human stem cell-derived neurons to amyloid plaque pathology in the brain of an amyloid mouse model (*App*^*NL−G−F*^*/Rag2*^*−/−*^). These neurons display pathological Tau accumulation and, interestingly, GVD-necroptosis activation. Temporal transcriptomic analyses of transplanted neurons unveiled that MEG3, a long noncoding RNA, acts as an upstream activator of the GVD-necroptosis pathway in this model. Most importantly, downregulating MEG3 expression using shRNA or inhibiting necroptosis using pharmacological or genetic approaches targeting RIPK1, RIPK3, or MLKL all effectively prevented neuronal cell loss, providing compelling evidence for the involvement of necroptosis in amyloid plaque-induced neuronal degeneration [11]. These observations strongly indicate a plausible role of the necroptosis pathway in neuronal cell death in AD.

Both in the postmortem and xenotransplantation studies, we noticed a remarkable association between necroptosis markers and GVD bodies. The abundant presence of this pathology is particularly intriguing as it is generally assumed that once a cell death pathway is initiated, cells undergo complete demise within 24 h [13, 142]. The presence of the necrosome complex within GVDs suggests, therefore, a biological checkpoint aimed at preventing the execution of necroptosis (Fig. 3).

While acknowledging activation of various types of programmed cell death in AD beyond necroptosis (caspase-3 activation (discussed above), pyroptosis [116, 169], ferroptosis [169], autophagy [121], and other mechanisms [56], necroptosis seems to be the predominant cell death pathway by which neurons degenerate in AD. Of course, there might be significant interplay among different cell death pathways in AD and in chronic degenerative disease in general, and further work will indicate whether PANoptosis [147] is a better concept than necroptosis to understand the underlying molecular and cell biology mechanisms of neuronal cell loss in AD.

## GVD, necroptosis, and Tau pathology

Simchowicz and Hirano described already an intraneuronal accumulation of aggregates in a membrane-bound vacuole (~ 3–5 µm diameter) with central granulated structures in AD more than 100 years ago (Fig. 1) [65, 78]. These structures are called GVD bodies or GVDs and are specific to neurons. GVDs were mainly observed in hippocampal pyramidal neurons in limbic and neocortical regions in various pathological conditions such as AD, PD, ALS, PSP, PSP, Pick’s, and Guam diseases and have become one of their histopathological features [163]. While GVDs are observed in healthy conditions as well, their abundance per neuron and the number of neurons with GVD significantly increase in pathological conditions [9, 10, 78, 163, 178]. In AD, similar to extracellular amyloid plaques and neurofibrillary tangles, GVDs also spread through the brain and reflect the spatiotemporal distribution of the neurofibrillary tangle pathology [83, 163].

Earlier research has shown that GVD lesions correlate with the degeneration of neurons in brain regions such as the entorhinal cortex, hippocampus, and, to some extent, the frontal cortex in AD and with accumulating pTau and Aβ pathology [83, 86, 91, 136]. Whether a causal relationship exists between GVD and neuronal loss remained unclear. Tau and GVD pathologies are frequently observed in the same neurons in the aforementioned vulnerable brain regions [44–46, 140, 177]. GVDs are thought to originate from late-stage autophagy structures and contain several aggregated proteins relevant to AD pathogenesis [49, 174]. Similar to pathological Tau, GVD first appears in the CA1 region of the hippocampus, and, like pathological Tau aggregates, accumulation of GVD is better correlated with neuronal cell loss than Aβ plaque pathology. Aβ plaques [16, 80, 153, 162, 172], being a disease-defining feature of AD, are not directly correlated to neuronal cell death or cognitive decline [16, 51, 79, 80]. They are likely acting as a trigger of a gradually developing chronic gliosis and neuroinflammation [153] acting upstream in the disease pathway, for instance by inducing MEG3 expression [11]. Aβ-pathology is also linked to both the formation and the spreading of pathological Tau and is in that way also linked to activation of necroptosis and GVD formation [14, 63, 163], in accordance with its role as a trigger, not a driver of the disease [79].

There is differential vulnerability among neurons to Tau, GVD pathology, and neurodegeneration [44, 95, 136, 140, 175]. Excitatory cholinergic basal forebrain neurons, as well as neurons in the entorhinal cortex, hippocampus, and subiculum, among other regions [45, 46], are particularly prone to degeneration. In contrast, inhibitory neurons are relatively spared from Tau pathology, although they are functionally affected early in the disease [44, 53, 95, 167]. Similarly, there is an inverse correlation between neuronal myelination and perineuronal nets with the onset of Tau pathology. Tau pathology tends to appear initially in cortical regions where myelination occurs later in development and spreads to areas with higher levels of myelination [23–25]. Moreover, neurons with aggrecan-based perineuronal nets or brain regions with abundant extracellular matrix chondroitin sulfate proteoglycans show less Tau and tangle pathology [27, 117]. We hypothesize that this selective vulnerability within neuronal subpopulations among different brain regions may contribute to discrepancies in Tau and GVD distribution.

A direct role of Tau in GVD generation was suggested in in vitro experiments using rodent primary cultures stimulated with Tau seeds, which induced GVD bodies exclusively in neurons but not in glia [174]. We have recently confirmed these and have observed that neurons deficient in Tau expression are not able to generate GVD in vitro (T’Syen, Balusu and De Strooper, manuscript in preparation). GVDs have been observed only in older Tau transgenic mice (24 months, e.g., Tau22, JNPL3, and PS19) and double transgenic mice carrying both human APP and Tau transgenes, but not in APP transgenic mice alone [70, 84, 86, 96, 113, 179]. Moreover, crossing APP transgenic mice with Tau transgenic mice accelerated Tau, GVD pathology, and neurodegeneration in line with an upstream role of amyloid in disease acceleration [79, 87, 96]. Intriguingly, inhibition of necroptosis using brain-penetrant small molecules in these mouse models rescued neuronal loss, confirming the direct role of necroptosis in AD-relevant neuronal loss [11, 87].

## Origin, content, and fate of GVD bodies and their association with Alzheimer’s disease pathogenesis

The mechanism of GVD formation and its relationship with pathological Tau in neurons remains poorly understood. Earlier studies using electron microscopy revealed a double-layer membrane, which suggested an autophagic origin [124]. However, recent immunohistochemical analysis of postmortem brain samples and in vitro modeling indicate the absence of LC3 and p62, both early autophagy markers, and EEA1, an early endosome marker [49]. GVDs exhibit, however, immunoreactivity to late autophagic markers like LIMP2 and LAMP1 on the outer membrane, as well as to endocytic markers such as CHMP2B in their dense core [49]. GVDs in mouse primary culture neurons induced by aggregated Tau are immune reactive to markers such as CK1δ, CK1ɛ, CHMP2B, and pPERK. The proteolysis reporter DQ-BSA is found in most GVDs, indicating that they contain degraded endocytic cargo and fuse with lysosomes [173, 174]. GVDs in cell culture, like their counterparts in vivo, exhibit immunoreactivity to LAMP1 and LIMP2, suggesting the contribution of lysosomes [49].

The cumulative observations in brain samples and in vitro models suggest that the GVDs harbor late-stage autophagy markers and amass at the nexus of autophagic and endocytic routes, probably as a consequence of an incomplete formation of autolysosome formation, which subsequently accumulates as GVDs [49].

The content of GVDs might provide further insights into their origin. Their dense cores contain coarse electron-dense protein aggregates, while the surrounding area appears floccular and liquid-like. Analytical EM studies revealed the presence of aluminum in GVDs [124]. A myriad number of proteins from different subcellular compartments accumulate in GVDs, including components of the unfolded protein response (UPR), other stress-related proteins, ubiquitin, neurofilament, kinases (GSK3β, CDK5, CK1α, CK1β CK1δ, MAPK, SYK, MARK 3&4, and JNK), disease-associated proteins (pTau, pAβ (Ser26), and pTDP43) [67, 89, 170], we refer to several excellent recent reviews for more exhaustive coverage of the proteome of GVD [67, 83, 173].

As several disease-associated proteins accumulate in GVD, there is great potential in further exploring the content of GVD for novel diagnostic markers of AD. CK1 is such an example. It is a biomarker for GVD in AD brains and CK1 levels can increase by up to > 20-fold in neurons containing tangles and GVDs [180]. In addition, members of the CK1 family are known to phosphorylate Tau, RIPK1 and RIPK3, components of the necrosome [61]. It is intriguing to see both CK1 and its substrates accumulating within the GVD subcellular compartment.

The exact role of GVD in the neurodegeneration process remains unclear. GVDs may potentially handle misfolded proteins. Tau is known to be degraded by autophagy and endocytosis [1] and might end up in late autophagic or GVD compartments if degradation is hampered. However, the question remains whether pathological Tau specifically triggers the necroptosis cell death pathway in neurons and whether other aggregates can induce similar pathology [37, 113, 176].

## GVD-necroptosis pathway: protective or detrimental

GVDs could serve as a defense mechanism against necroptosis, or they might reflect a gradual, slow-acting form of this process. Several cell-type-specific checkpoint mechanisms have been identified that are capable of reviving cells from necroptotic demise, and it might be that the GVD accumulation is just reflecting one of those mechanisms (Fig. 3) [41, 141, 185]. Instead of permeabilizing the cell membrane and executing necroptosis, membrane-bound pMLKL can be endocytosed via a flotillin-mediated mechanism and degraded in the lysosomes (checkpoint 2, Fig. 3). Alternatively, ALIX-syntenin-1-mediated exocytosis of pMLKL via extracellular vesicles can also protect the cell membrane (checkpoint 1, Fig. 3) [41, 181, 185]. It looks like the neurons in AD, maybe because of an altered endo-lysosomal system, may accumulate such vesicles with aggregated pMLKL and potentially other toxic aggregates, thereby contributing to the formation of GVDs [1, 41, 158, 181, 185]. Notably, genetic studies, in particular genome-wide association studies (GWAS) have identified several risk genes of AD which are operating in the endo-lysosomal network (ELN), such as PICALM, PLD3, BIN1, CSTD, CLU, UBQLN1, GRN, and SORL1 [12, 15, 74, 109].

## Endo-lysosomal autophagy is disturbed by Tau pathology and provides links to GVD-necroptosis

In the context of AD, the intracellular aggregates of hyperphosphorylated, conformationally altered Tau are associated with synaptic loss, GVD formation, necroptosis activation, and neuronal loss [19, 72, 86, 136, 174, 182]. Tau is an intrinsically disordered protein and is mainly present in axons. Post-translational modifications such as phosphorylation, acetylation, and ubiquitinylation negatively impact its ability to interact with microtubules, leading to aggregation. The aggregated Tau is either degraded or assembled into filamentous inclusions, which vary among the different Tauopathies [43, 148, 171].

The question of whether pathological inclusions of Tau are only indicators of disease progression or directly involved in cellular demise remains unresolved. Conflicting evidence from various model systems used to model AD Tau pathology has led to ongoing controversy regarding whether monomers, oligomers, or filaments, are toxic [120]. Recent data from in vitro and in vivo studies using pathological Tau seeds indicate that these seeds can be internalized and can propagate pathology both in vitro and in vivo [14, 30, 63, 93, 108]. These toxic protein aggregates in the cytosol are degraded via either the proteasome or autophagic-lysosomal systems and we speculate that disturbances of these pathways, either upstream of Tau or caused by accumulating Tau, are linked to GVD and necroptosis.

The ubiquitin–proteasome system (UPS) marks Tau for degradation via monoubiquitylation or polyubiquitination [33]. Unlike monoubiquitination, specific branching patterns of polyubiquitination at amino acid residues (K6, K11, K48, K63 and M1) determine the route of protein degradation. K48-linked polyubiquitination is predominant and targets the Tau protein to the proteasome. K63-linked polyubiquitination can serve diverse functions, including directing Tau to autophagic or lysosomal pathways, but it could also promote the formation of insoluble inclusions and facilitate endocytosis [33]. Polyubiquitination could indeed contribute to Tau accumulation within GVDs, particularly in the presence of autophagy defects [31, 33, 171].

Strong arguments for the involvement of proteins belonging to the endo-lysosomal network and autophagy in AD pathogenesis came from the GWAS studies [15, 74]. Impairment in the auto-lysosomal axis in neurons has also been documented in AD brains and transgenic mouse models [132]. For example, an increase in the expression of RAB5 and RAB7 has been observed exclusively in regions prone to degeneration in individuals with mild cognitive impairment (MCI) and AD [55]. The activity of the mammalian target of rapamycin (mTOR), a negative regulator of autophagy, is notably elevated in AD brains, which also positively correlates with Braak staging [4, 132].

Interestingly, the accumulation of autophagosomes in the dystrophic neurites around the plaque has been noted in AD brains as well as in several transgenic amyloid mouse models such as APP-PS1, 5xFAD, and App^NL−G−F^ [146]. PLD3, also known as phospholipase D3, has been linked to late-onset AD (LOAD) and is highly enriched in the dystrophic neurites both in human AD brains and transgenic amyloid mouse brains [42, 118, 143, 184]. Functional experiments with overexpression of PLD3 caused endolysosomes to enlarge, leading to their accumulation and a decline in axonal conduction. Conversely, the deletion of PLD3 reversed these abnormalities, thereby establishing a mechanistic link between PLD3 expression and the enlargement of endo-lysosomal compartments in AD. This adds to the substantial evidence suggesting that AD pathology disrupts autophagy and the endo-lysosomal system [42, 118, 143, 146, 184].

GVDs, which are late-phase autolysosome compartments, accumulate in the soma of degenerating neurons. They contain a diverse range of disease-associated proteins such as pTDP43 and pTau [164, 170]. Increased expression of early autophagy markers such as MAP1LC3B-II and p62 (also known as SQSTM1/p62) is associated with neurofibrillary tangles [92, 134]. Whether the increase in expression of autophagy markers in AD represents a high demand for autophagy as a protective reaction or an impaired autophagosome maturation in neuronal cell bodies as part of the pathogenesis is unclear. The lysosomal acidification system, downstream of autophagosome maturation, is crucial in effectively breaking down and recycling luminal contents [81, 122]. In AD, reduced expression of lysosomal proteins like Cathepsin D (CTSD) and lysosomal-associated membrane protein 1 (LAMP1) and subcellular mis-localization are potentially reflecting a hampered lysosomal acidification in both neurons and glia [69, 77, 122].

It is intriguing that proteins detected in autophagosomes within dystrophic neurites resulting from amyloid plaque deposition are also present in the GVDs [67], which are primarily triggered by intracellular Tau pathology. Despite the shared proteome composition between dystrophic neurites and GVD compartments, there are significant differences in the maturation and intracellular fate of these organelles. For instance, early-stage immature autophagosomes in axonal DNs undergo retrograde transport towards the cell body, where they subsequently fuse with lysosomes for further degradation [66]. However, the significant accumulation of both immature and mature autophagic vesicles within DNs compared to neuronal perikarya suggests either impaired retrograde transport [18, 122, 159] or a more effective and robust autophagic process, specifically within the axonal dystrophic neurites [2, 122, 130]. Furthermore, senescence and impaired autophagy can result in the accumulation of intracellular Aβ, potentially contributing to downstream effects involving Tau, necroptosis, and GVDs [3, 57, 82, 155] (Fig. 2).

## Canonical necroptosis cell death pathway and relevance to Alzheimer’s disease

The intermediate domain of RIPK1 contains a RIP homotypic interaction motif (RHIM), which facilitates both homo- and heterodimeric interactions with other RHIM-containing proteins, including RIPK3, Toll/IL-1R domain-containing adapter-inducing interferon-β (TRIF), and Z-DNA binding protein 1 (ZBP1) [35, 58, 119]. These RHIM motif domains are pivotal in initiating necroptosis. Furthermore, the C-terminal death domain (DD) of RIPK1 also mediates both homo and heterodimerization with other intracellular death domain-containing proteins like Fas-associated protein with a death domain (FADD), TNFR1, and FAS [58, 166]. RIPK1 can, on the contrary, prevent cell death by regulating pro-survival B-cell lymphomas-2 (BCL-2) and X chromosome-linked inhibitor of apoptosis (XIAP) and inflammatory gene expression in the cells (Fig. 3) [35]. RIPK3 is another core component of the necroptotic cell death pathway. Upon phosphorylation, either by RIPK1 or through self-phosphorylation, RIPK3 recruits MLKL and triggers MLKL phosphorylation [166]. Like RIPK1, RIPK3 also contains a RHIM domain that enables the formation of a signaling complex between RIPK1 and RIPK3, characterized by amyloid-like structures formed by the two proteins, thereby initiating downstream signaling events [115]. RIPK3 can be activated by ZBP1, a nucleic acid pattern recognition receptor that binds to cytosolic z-DNA or z-RNA. MLKL, finally, is the “execution protein” of the necroptosis pathway. MLKL consists of a four-helical bundle (4HB) domain at the N-terminal and a pseudokinase domain at the C-terminal side [131]. RIPK3 facilitates the phosphorylation of MLKL, leading to a conformational change in its structure. This alteration induces MLKL aggregation, necrosome formation, translocation to the cell membrane, membrane permeabilization, and ultimately leads to cell death [58, 166].

Classically, necroptosis has been studied in the context of inflammatory stimuli such as tumor necrosis factor (TNF). TNF binds to tumor necrosis factor receptor 1 (TNFR1), initiating the recruitment of cellular inhibitors of apoptosis proteins (cIAP), RIPK1, TNF receptor-associated factor (TRAF), and TNF receptor-associated death domain (TRADD) to the intracellular domain of TNFR1, resulting in the assembly of complex I and induction of a pro-inflammatory response [129]. TNFR1 prompts apoptosis via complex IIa (Fig. 3). However, in conditions where apoptosis is deficient, TNF triggers caspase-independent cell death through RIPK1 (complex IIb) [36].

Given the strong link between TNF and necroptosis, it becomes imperative to inquire whether TNF, interferon, or Toll-like receptor (TLR) ligands—known stimulants of necroptosis—are upregulated in AD. While we provide a summary of previous research, it appears imperative to undertake further investigations in this area, as correlative evidence indicates its potential significance. Inflammatory conditions such as rheumatoid arthritis (RA), psoriasis, and inflammatory bowel disease, where TNF plays a significant role, are associated with a higher likelihood of developing AD [29, 135, 138, 187]. While epidemiological evidence provides correlative evidence of an association between anti-TNF treatment in RA and a lower incidence of AD, this relationship does not prove, of course, causality [187]. A single nucleotide polymorphism in TNF (G308A; rs1800629) correlates with susceptibility to AD in the Chinese population, whereas the same SNP shows a protective effect in the European population [6, 20, 138, 168].

Reports measuring TNF or TNFR1 in patients are also not unequivocal [135, 157, 160]. One potential source of TNF in the brain is the microglia. Microglia exhibit a diverse range of cellular states when exposed to amyloid Aβ pathology, including the cytokine response microglia or CRM characterized by upregulation of a whole series of pro-inflammatory cytokines [105, 106]. One study reported a close association of the activated HLA-DR+ microglia and CD8+ T cells in close proximity to neurons expressing pMLKL. However, it is not clear whether microglia locally produce TNF to initiate necroptosis in neurons [75].

## Exploring therapeutic strategies by targeting the necroptosis pathway in Alzheimer’s disease

The recent approval of Aβ-targeting immunotherapy and progress in treating Tau pathology are hopeful developments in the field of AD [40]. However, removing the biochemical hallmarks of the disease at the stage that dementia symptoms occur will not be enough to stabilize the disease. Moreover, it takes about 1 year to clear amyloid plaques from the brain [151], and likely a similar time for Tau. During treatment, neurons will continue to suffer from amyloid stress, and necroptosis will be maintained. To maximize therapeutic benefits, it might be essential to stop neuronal loss while the neuroinflammatory environment induced by amyloid plaques gradually resolves. Therapeutically inhibiting the main cell death mechanisms while Aβ therapy is building up its benefits might result in a better outcome for the patient.

Several necroptosis inhibitors are approved already or are in various stages of development for the treatment of cancer. For instance, ponatinib (targets both RIPK1 and RIPK3) and dabrafenib and Sorafenib (targets RIPK3) were approved for the treatment of leukemia [7, 111]. These kinase inhibitors are not specific and known to target non-overlapping kinases such as SRC, ABL, BRAF, RAF, VEGFR, PDGFR, FGFR, KIT, SIK1, NEK11, RET, TIE2, BCR-ABL, EPHR, FLT3, TAK1, and RIPK2 [110]. We recently demonstrated that ponatinib and dabrafenib could rescue neuronal loss in both the preclinical xenograft model of AD as well as in a mouse model harboring both amyloid and Tau pathology (double transgenic mice, APP23xTau58) [11, 87, 119].

Nearly 37 compounds are being studied to target RIPK1, the upstream kinase in the necroptosis cascade with 27 in preclinical stages and 10 in clinical trials. In 2018, Denali forged a partnership with Sanofi aimed at developing CNS-specific RIPK inhibitors for AD, ALS, and MS indications. DNL788 (SAR443820) is a selective and potent RIPK1 inhibitor succeeding DNL747, which Denali and Sanofi halted after Phase 1 due to apprehensions regarding its long-term toxicity. DNL788 was tested in the clinic for ALS and MS indications. In the ALS Phase 2 HIMALAYA trial the drug failed to meet the primary endpoint, i.e., a better functional outcome as measured with the ALSFRS-R (ALS Functional Rating Scale—Revised). The role of necroptosis in ALS remains a subject of ongoing investigation. Some studies suggest that necroptosis is implicated in the degeneration of motor neurons [71], while others, using preclinical models like the SOD1 model of ALS, have reported no alteration in motor neuron degeneration upon deletion of RIPK1 [39]. A recent neuropathological study revealed no accumulation of the necrosome complex in the central cortex and spinal cord [144]. Another RIPK1 inhibitor, SIR-2446 is an oral RIPK1 inhibitor being developed by Sironax therapeutics for the treatment of AD and MS and is currently in Phase 1 clinical trials. DNL788 is also being tested in relapsing–remitting MS in a Phase 2 in collaboration with Sanofi (Table 1).

**Table 1:** List of drugs targeting the necroptosis cell death pathway in CNS-related conditions

Drug	Target	Company	Phase	Indication
Necrostatin-1	RIPK1	Academic/non-profit	Preclinical	AD, PD, ALS
7-Cl-O-Nec-1	RIPK1	Academic/non-profit	Preclinical	AD, PD, ALS
BSC-3301	RIPK1	BisiChem	Preclinical	AD, MS, ALS
SIR-2446	RIPK1	Sironax Ltd	Phase 1	AD, MS
AMX0035	RIPK1	Amylyx Pharmaceuticals	Phase 3	ALS
DNL788/SAR443820	RIPK1	Denali Therapeutics	Phase 1	ALS (ceased), MS
VRN-04-1 (VRN-04)	RIPK1	Voronoi	preclinical	Inflammation
GSK’843	RIPK3	GlaxoSmithKline	Preclinical	Neurodegenerative diseases (broad)
IRP-1529	RIPK3	IRP Systems	Preclinical	AD, PD
PN10943	RIPK3	ProteoNic	Preclinical	AD, PD

Currently, there are no drugs in the clinical phase of development specifically aimed at targeting RIPK3, although some are in the preclinical pipeline. Compounds such as GSK’840, GSK’843, and GSK’872 that target RIPK3 induced a conformational change, enabling the recruitment of RIPK1 through the RHIM domain, resulting in caspase 8 activation and the initiation of apoptosis [107]. This unexpected induction of apoptosis by RIPK3 upon drug binding has impeded drug discovery programs for RIPK3. Recent studies have shown that Heat shock protein 90 (HSP90) regulates the necroptosis pathway by targeting RIPK3 and MLKL in a classical TNF induced necroptosis model [73, 98, 186], which might open the possibility of targeting RIPK3 or MLKL via HSP90 inhibition [97]. However, it remains to be established whether similar mechanisms are conserved in the context of GVD-necroptosis in AD.

Alternatively, one could study and target the upstream regulators of the necroptosis pathway, especially if they are specific for AD. For instance, the long noncoding RNA, MEG3, is differentially regulated in AD and has been shown to regulate cell death pathways [11, 59, 99, 101]. In vitro overexpression of MEG3 in neurons can activate the necroptosis pathway and its effect can be countered using ponatinib, dabrafenib, or necrosulfonamide [11]. Likewise, inhibition of MEG3 expression in transplanted neurons rescued neuronal loss, consistent with observations made in vitro. While the precise mechanism by which MEG3 induces necroptosis and the upstream factors that induce MEG3 expression remains unclear, it is intriguing to speculate that identifying those might finally provide a molecular link between Tau pathology and neuronal loss. Inhibiting such upstream necroptosis-inducing factors might present a broader opportunity to target this pathway and specifically inhibit neuronal loss in AD.

## Conclusion

Demonstrating that necroptosis is involved in Tau pathology-driven neuronal loss in AD seems a pivotal observation. This observation provides the foundation for further work aimed at linking those pathologies at the molecular level and identifying kinases or other proteins that connect neuroinflammation, Tau pathology, the induction of the necrosome, and components of the GVD in a consistent pathway that can be targeted for the treatment of AD. Similarly, unraveling the role of upstream triggers of this pathway (Aβ and/or inflammatory mediators) seems a crucial aim for further Alzheimer’s research. A critical note remains that cell death pathway molecules are multifunctional and can be involved in an array of mechanisms leading to protection or cell death. The following years will teach to what extent necroptosis is necessary and sufficient for neuronal loss in AD or whether it is the culmination of varying cell death pathways and PANoptosis that determines the outcome of this devastating disease [161].

## Abbreviations

AD: Alzheimer’s disease
ALS: Amyotrophic lateral sclerosis
APP: Amyloid precursor protein
ARTAG: Aging-related tau astrogliopathy
Aβ: Amyloid beta peptide
BCL-2: B cell lymphoma-2
cIAP: Cellular inhibitors of apoptosis proteins (cIAP)
DD: Death domain
DNs: Dystrophic neurites
ELN: Endo-lysosomal network
ESCRT: Endosomal sorting complexes required for transport
FADD: Fas-associated protein with a death domain
GVDs: Granulovacuolar degeneration
LOAD: Late onset Alzheimer’s disease
MLKL: Mixed lineage kinase
MS: Multiple sclerosis
NDDs: Neurodegenerative diseases
PCD: Programmed cell death
PD: Parkinson’s disease
pTAU: Phosphorylated Tau
RA: Rheumatoid arthritis
RHIM: RIP homotypic interaction motif
RIPK1: Receptor-interacting serine-threonine protein kinase 1
RIPK3: Receptor-interacting serine-threonine protein kinase 3
TLR: Toll-like receptor
TNF: Tumor necrosis factor
TNFR1: Tumor necrosis factor receptor 1
UPR: Unfolded protein response
UPS: Ubiquitin proteasome system
ZBP1: Z-DNA binding protein 1
